# Computational Methods for Metabolomic Data Analysis of Ion Mobility Spectrometry Data—Reviewing the State of the Art 

**DOI:** 10.3390/metabo2040733

**Published:** 2012-10-16

**Authors:** Anne-Christin Hauschild, Till Schneider, Josch Pauling, Kathrin Rupp, Mi Jang, Jörg Ingo Baumbach, Jan Baumbach

**Affiliations:** 1 Computational Systems Biology Group, Max Planck Institute for Informatics, D-66123, Saarbrücken, Germany; 2 Cluster of Excellence for Multimodal Computing and Interaction,Saarland University, D-66123 Saarbrücken, Germany; 3 Department Microfluidics and Clinical Diagnostics, KIST Europe-Korea Institute of Science and Technology Europe, Campus E7.1, D-66123, Saarbrücken, Germany; 4 Computational Biology group, Department of Mathematics and Computer Science, University of Southern Denmark, DK-5230, Odense, Denmark

**Keywords:** ion mobility spectrometry, clinical diagnostics, peak detection, statistics, statistical learning methods, metabolomics, volatile organic compounds

## Abstract

Ion mobility spectrometry combined with multi-capillary columns (MCC/IMS) is a well known technology for detecting volatile organic compounds (VOCs). We may utilize MCC/IMS for scanning human exhaled air, bacterial colonies or cell lines, for example. Thereby we gain information about the human health status or infection threats. We may further study the metabolic response of living cells to external perturbations. The instrument is comparably cheap, robust and easy to use in every day practice. However, the potential of the MCC/IMS methodology depends on the successful application of computational approaches for analyzing the huge amount of emerging data sets. Here, we will review the state of the art and highlight existing challenges. First, we address methods for raw data handling, data storage and visualization. Afterwards we will introduce de-noising, peak picking and other pre-processing approaches. We will discuss statistical methods for analyzing correlations between peaks and diseases or medical treatment. Finally, we study up-to-date machine learning techniques for identifying robust biomarker molecules that allow classifying patients into healthy and diseased groups. We conclude that MCC/IMS coupled with sophisticated computational methods has the potential to successfully address a broad range of biomedical questions. While we can solve most of the data pre-processing steps satisfactorily, some computational challenges with statistical learning and model validation remain.

## 1. Introduction

Ion mobility spectrometers combined with a multi-capillary column (MCC/IMS) are well known for detecting volatile organic compounds (VOCs). Initially developed for military purposes, nowadays they are used for various applications: process control in chemical or petro industry or scanning human exhaled air/breath, bacterial colonies or cell lines for example. The combination of the MCC/IMS methodology and sophisticated computational approaches has the potential to successfully address a broad range of biomedical questions. On the one hand, building statistical models for disease prediction and identification of biomarkers, and on the other hand, determining cell states and metabolic responses of microorganisms or the assessment of food quality.

### 1.1. Overview: Ion Mobility Spectrometry

There are several analytical detection methods for human breath investigations. The major spectrometric methods currently employed are gas chromatography-mass spectrometry (GC/MS) [1,2,3,4], solid phase micro extraction-gas chromatography coupled with mass spectrometry (SPME-GC/MS) [1,5,6], electronic noses [7,8,9,10], proton transfer reaction-mass spectrometry (PTR-MS) [11,12] and ion mobility spectrometry (IMS) [13,14,15,16,17,18,19]. The real time analysis (e.g. PTR-MS, IMS) has the advantage that no pre-concentration step is needed [20]. Sampling is achieved directly by using Tedlar bags [21,22,23], needle traps [24], SPME [5,25], sample loops [26] and different adsorbents. They are all non-invasive and should provide early and fast diagnosis or therapy monitoring for the identification of disease-specific biomarkers in the patients’ breath.

Ion mobility spectrometry is a method to detect volatile organic compounds (VOCs). The first IMS instruments, created in the early 1970s, were originally used for military applications [27,28]. Further IMS were used to detect drugs or explosives, e.g. at airports. With the growing importance of metabolomics, the focus changed, and today IMS is also used in medical applications. Through combination of the IMS with multi capillary columns (MCC), many possible application opportunities arise. The main advantages of this method are the short time required to collect a sample (about 10 s), the non-invasive nature of the method, the use of easily obtainable exhaled breath, and the robust and easy handling in every day practice. The MCC/IMS based on BioScout was developed by B&S Analytik (Dortmund, Germany) for medical [29,30] and biomedical [31] applications as well as for process analysis [14].

**Figure 1:**
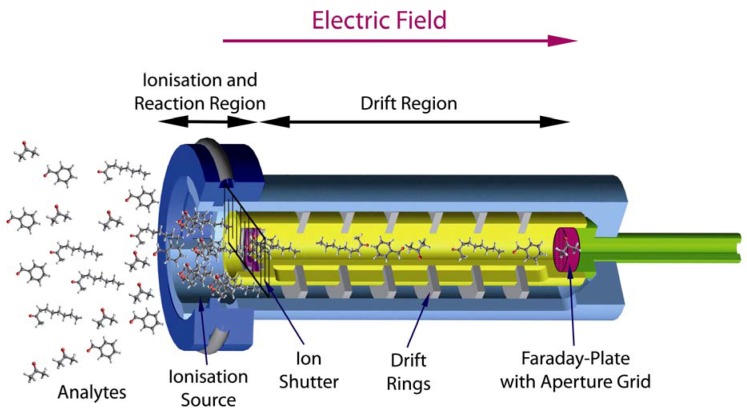
Working principle of an Ion Mobility Spectrometer.

The time needed to acquire a single spectrum takes only 10 ms to 100 ms [32]. To receive a MCC/IMS chromatogram a certain setup of the MCC/IMS is needed. Driven by the carrier gas the analytes first reach the MCC, where the pre-separation takes place. In this column there are approx. 1.000 parallel capillaries, each with a film thickness of 200 nm and an inner diameter of 40 µm. In general the OV-5 phase (5% Phenyl / 95% Dimethyl Polysiloxan) is used [30]. After passing the MCC, the analytes reach the ionization chamber, where they become chemically ionized by collisions with ionized carrier gas molecules. The carrier gas molecules were previously ionized by a radioactive ionization source (^63^Ni) and from so-called reactant ions. After the chemical ionization, the resulting product ions enter the drift region when the ion shutter is open (Figure 1). In this region, an external electric field is applied. A so-called drift gas will flow from the Faraday-Plate towards the ions; neutral molecules cannot enter the drift region and the ionized molecules will gain energy from the electric field and soon reach a steady drift velocity by collision with neutral drift gas molecules. That means that all molecules are, in an ideal case, totally separated when they reach the Faraday-Plate. In the end, a spectrum is generated, which is called ion mobility spectrum. The accumulation of all IMS spectra pre-separated by the MCC is called IMS chromatogram.

### 1.2. Outline

**Figure 2:**
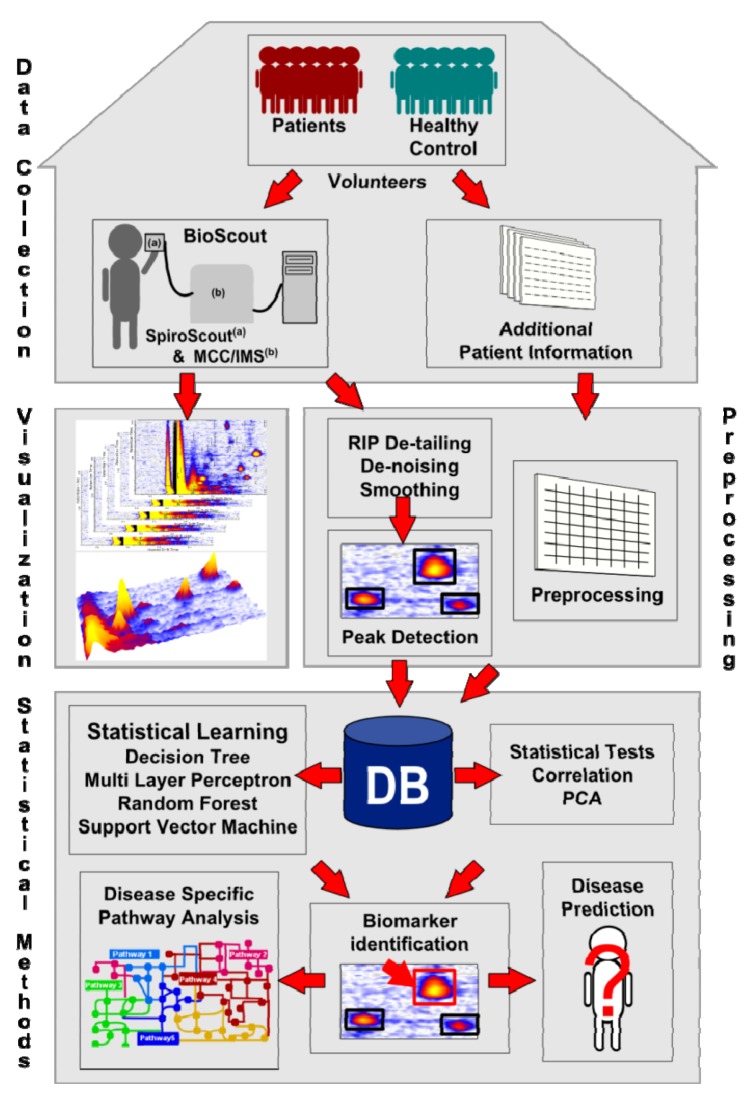
Workflow of the data processing, data mining and evaluation methods used in clinical breath diagnostics.

The present paper gives an overview of the state of the art data processing, data mining and evaluation methods, used for the analysis of MCC/IMS chromatograms. Figure 2 depicts the workflow of the processing pipeline handling the MCC/IMS data. The first step is the data collection in laboratories and hospitals, both the results of the diagnostic technique analyzing human breath, as well as additional information (e.g., age, gender, medication, medium of the bacterial strain, various diagnostic techniques, *etc*.). The next step is the pre-processing of the chromatograms, enhancing the quality (e.g., de-noising, smoothing) and detecting the VOC areas (peaks). The results are verified using the available visualization tools. The pre-processed data is subsequently accumulated in a centralized data repository, e.g., a database. Furthermore, the additional information of the organism is prepared to be included into the system. Statistical techniques like Mann-Whitney U test and principal component analysis as well as statistical learning methods, e.g., decision tree and support vector machines are applied to find biomarkers. The biomarkers are verified in the wet lab. They may later be used for disease prediction and disease specific pathway analysis. 

Each step of the workflow will be explained in the following chapters of this review. We start with a recap of the data format, the visualization and a detailed explanation of the different preprocessing steps: RIP detailing, smoothing, de-noising and peak finding. In addition, an introduction to the existing databases and the future requirements is given. Section three depicts the studies using statistical techniques like Mann-Whitney U test, correlation and principle component analysis. We continue with a description of the statistical learning methods applied to MCC/IMS data sets in section four. Finally, we will sum up, illuminate unsolved problems, and provide potential solutions.

## 2. First Steps with IMS Data

### 2.1. Data Format

Over the years, the central question that has motivated the application of IMS and MCC/IMS devices has changed from “Is a particular analyte present?” (detection of explosives, drugs, and chemical warfare agents) to “Which analytes are present in which concentration?” (biomedical on-site analysis, drug monitoring, disease fingerprinting) [33]. This leads to utilizing computational and bioinformatical techniques, which are explained throughout the following sections. A broader area of application comes with new requirements in terms of tracking experimental conditions, since this may influence not only the measurement itself, but also its interpretation and give vital information for further data processing, such as the combined analysis of data from different studies. Thus, a sophisticated uniform data format is required, which stores not only the data itself but also experimental and technical conditions. Vautz *et al.* proposed such a standard file format [33]. It consists of a header and the data matrix. The header comprises all sampling conditions such as general information, sample information, IMS (device) information, external sampling control, and statistics. They also provide standard nomenclature rules and an extension that is dedicated to sensor-controlled sampling. For details, please refer to supplementary material of Vautz *et al.* [33]. A later, related publication by Maddula *et al.* suggests an extension to the standard file format, which allows cross-linking gas chromatography/mass selective detector (GC/MSD) data with MCC/IMS data [34].

### 2.2. Visualization

**Figure 3:**
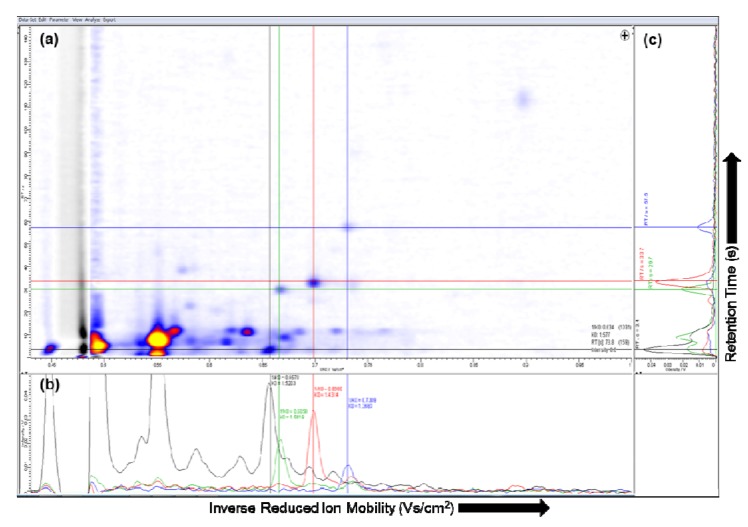
**(a)** Visualization of the ion mobility spectrometry (IMS)-chromatogram; **(b)** Single ion mobility spectrum; **(c) **Single multi-capillary column (MCC) spectrum.

Several software tools are available for the visualization of IMS-chromatograms. The software package IPHEX (by A. Bunkowski, University Bielefeld, Germany) supports the visualization including single spectra and total ion current of the MCC [35]. The commercial software package VisualNow (B&S Analytik, Dortmund, Germany), which is implemented in Java, is another state of the art software tool. It provides the ability to show two- and three-dimensional plots of the whole IMS chromatogram as well as all technical parameters. The acquired data of the MCC/IMS file includes a set of parameters describing the measurements, experimental setup and a set of single spectra at different retention times, see Section 2.1 for more details [36]. In both software tools the IMS-chromatogram of the selected data file is plotted as a two-dimensional picture, e.g., VisualNow plot shown in Figure 3 (a). 

In the MCC/IMS-chromatogram the X-axis represents the reduced inverse mobility 1/Ko (Vs/cm^2^) and the Y-axis shows the retention time (s). The reduced inverse mobility is proportional to the drift time. Moreover, in order to compare spectra obtained using different experimental conditions the value is normalized by temperature and pressure [37]. The signal height is the signal from the Faraday plate of the IMS device. In general, the so-called intensity is color-coded in both plots, whereby the yellow color means the highest signal and the white color the lowest. In the three-dimensional plot the Z-axis expresses the intensity [38]. In order to compare or show single spectra of different peaks, a spectrum can be selected and shown in a separate plot, which can be examined visually.

**Figure 4:**
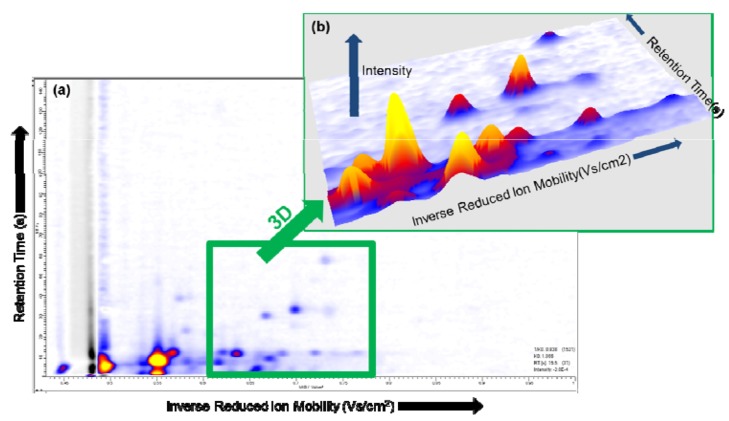
**(a)** IMS chromatogram; **(b)** A selected area (green) within the MCC/IMS chromatogram is converted into a three-dimensional plot.

The single spectrum at the selected ion mobility and the single spectrum at the selected retention time in VisualNow are shown in Figure 3 (b) and (c), respectively. Depending on the characteristics of the data, in some cases a three-dimensional plot can be suitable to identify and compare the peaks captured by the MCC/IMS [36]. A region in the two-dimensional plot can be selected, and visualized in a three-dimensional plot, see Figure 4 (a) and (b), respectively. 

In addition to this, both tools are capable of visualizing a mapping of the MCC retention time to the retention time of a gas chromatographic measurement. For details on the alignment methods between MCC and GC, we refer to [2] and [35].

### 2.3. Pre-processing

**Figure 5:**
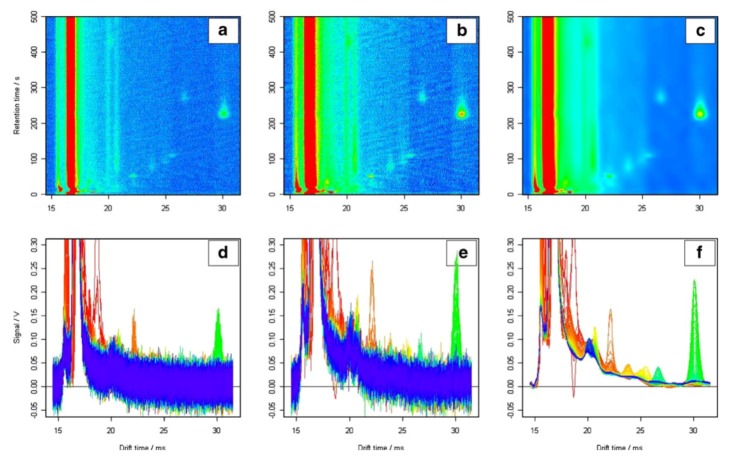
MCC/IMS chromatograms of raw **(a)** smoothed, **(b)** and de-noised, **(c)** data, illustrating the remaining information after de-noising and smoothing. 2D side views of raw **(d)** smoothed, **(e)** and de-noised chromatograms also show different baselines of the peaks caused by RIP tailing; **(f)** [41]. Reproduced with permission from Bader *et al.*, International Journal of Ion Mobility Spectrometry published by Springer-Verlag, 2008.

A MCC/IMS measurement typically consists of more than a million three-dimensional data points indicating signal intensities. Furthermore, there is a characteristic signal structure found in all IMS spectra of a chromatogram that is known as the reactant ion peak (RIP). The RIP is generally the highest and broadest peak and appears in the shape of a broad vertical line on the chromatogram (Figure 5). The signal descent on the right side of the RIP is called RIP tailing (Figure 5). RIP tailing can be considered as a source of disturbance. For this reason, Bader *et al.* performed RIP de-tailing by fitting a lognormal function to the mean of all spectra and subtracting this function from each spectrum in the chromatogram [39]. Bunkowski achieved RIP de-tailing by subtracting the 25% quantile intensity determined for each 1/K0 value over all spectra [40].

Besides RIP tailing, the random fluctuation in the signals, casually called noise, affects the ability to distinguish low intensity signals. Basically, all signal and non-signal (background) parts of a spectrum are overlaid by noise. Therefore, the data needs to be pre-processed by smoothing and de-noising methods (Section 2.1.3), in order to improve the signal-to-noise ratio as well as the clarity of the peaks, which are related to specific analytes. As the noise in the IMS data does not typically vary around zero, a baseline correction is performed, which improves the comparison of IMS data. Bader addressed this problem by subtracting the mean intensity of a pure noise region from all spectra of an IMS chromatogram [39].

#### 2.3.1. De-noising and Smoothing

In Bader *et al.* 2008 [39], a multi resolution analysis was applied to the data, which includes discrete wavelet transforms on different levels of resolution for both de-noising and smoothing, based on a combination of the approaches presented by Urbas *et al.* [42], and Cai *et al.* [43]. The corresponding wavelet coefficients, utilized to reconstruct the original chromatogram, can be altered using hard and soft thresholding. Removing coefficients corresponding to high frequency regions, independent of the coefficient amplitude, results in smoothing. The elimination of low amplitude coefficients regardless of frequency, results in de-noising. In doing so, IMS data is compressed to 25% or less of the original data, with negligible loss of information.

Another approach for de-noising is the application of a filter pipeline described by Bunkowski [40], where a median filter is first employed for de-noising, followed by a Savitzky-Golay filter, described in Savitzky *et al.* [44], and finally a Gaussian filter [45]. 

In addition to computational methods for de-noising, which are applied to the raw data, there are also methods for de-noising in electrical engineering, which are considered during the instrument design. Unfortunately all these methods, which are out of the scope of this publication, have the limitation of partial irrecoverable raw data loss. Especially in cases of large concentration differences within one sample and co-existing large, small, and sometimes overlapping peaks, the balance between noise reduction and the potential loss of resolution has to be considered carefully.

#### 2.3.2. Peak Detection

After pre-processing, peaks have to be identified in each single MCC/IMS measurement. Bader and Bunkowski developed different strategies to accomplish this task. Bader *et al.* presented three methods for peak finding referred to as Merged Peak Cluster Localization (MPCL), Growing Interval Merging (GIM), and Wavelet-Based Multiscale Peak Detection (WBMPD) [39]. The algorithms of Bunkowski *et al.* are based on water shed transformation (WST) [40]. The methods are explained in the following sections.

**Merged Peak Cluster Localization (MPCL):** This peak picking method utilizes a robust, locally weighted regression and smoothing scatterplot (LOWESS) algorithm by Cleveland *et al.* for baseline correction and reduction of RIP tailing [46]. In the first phase, all data points starting after the RIP are separated into two classes (peak and non-peak) by k-means clustering using a Euclidean distance metric of the intensity, whereby the two starting mean values can be chosen. Under the assumption of noise varying around zero, the non-peak class mean can be chosen as zero. Due to a large degree of misclassifications of high noise intensity points to the peak cluster, a filtering step is performed, whereby a peak cluster point is assigned to the non-peak cluster if at least one of its eight neighbor points belongs to the non-peak cluster. In the second phase, different peaks are identified by a merging regions algorithm based on Bruce *et al.* [47]. Taking the binary data points delivered from the first phase, adjacent points of the peak class are merged to form one peak. Finally, a set of distinct peaks for a single measurement is obtained. The limitation of this approach lies in the distinction of two overlapping neighbor peaks, where the overlap’s signal intensity is above the peak-to-noise-threshold. In this case, the merging regions algorithm will fail to distinguish between the two peaks [39].

**Growing interval merging (GIM): **This approach was used to overcome the resolution problems of the previous method. An iterative algorithm starts at the top of the intensity range, separates noise from peak data points and merges the discovered peaks in a stepwise manner along the intensity scale. Noise and RIP thresholds are defined by analysis of the intensity histogram, which is divided into three regions, namely noise (most data points), peak (second most data points) and RIP (small number of data points). The intensity range given in the peak region is divided into subintervals with evenly distributed data points. Those subintervals are scanned stepwise with descending intensity. Subsequently, the data points of the current sub-interval are assigned to the peak class. Afterwards, the second phase of the MPCL method is applied. This process yields peak lists with distinct peaks. All elements of the current peak list are merged with the ones contained in the previous peak list, provided they have several data points in common. Subsequently, the old peak parameters (maximum intensity with 1/K_0_ and RT coordinate, two ellipse axes and ellipse area) are overridden by the current parameters. This merging procedure allows the algorithm to distinguish between peaks which overlap or whose in-between area lifts off from the noise. Overall, GIM outperforms MPCL in terms of peak resolution, and extracts six parameters for each peak found. Nevertheless, there are limitations to the method, as overlapping peaks cannot always be distinguished. Furthermore, the separation of peaks with one or more local maxima in the tailing region is not possible.

**Wavelet-based multiscale peak detection (WBMPD): **The third peak-finding method of Bader [41] is partly based on “The multiscale processing of single spectra”, introduced by Randolph and Yasui [48], which is less suited for processing a series of spectra. Multiple resolution analysis (MRA) is performed using the maximal overlap discrete wavelet transform (MODWT), allowing the sample size to be arbitrary while the discrete wavelet transform is bound to a sample size that is a power of two. Furthermore, MODWT is translation invariant, which is beneficial for shifted IMS chromatograms. Compared to Daubechies D(4) wavelets, Haar wavelets show less artifacts in the RIP region at low retention times in a chromatogram, and are therefore preferred. The stepwise MRA process passes a chromatogram from higher to lower resolutions. It splits the low pass part of the current chromatogram into four sub-images: lowpass-highpass (LH), lowpass-lowpass (LL), highpass-lowpass (HL), and highpass-highpass (HH). The LH image of this so-called wavelet decomposition contains only details, which means the fine-grained variations within the chromatogram, in contrast to the coarse chromatogram structure of the LL image. Here, the GIM method of the previous paragraph is applied to the LH image. All decomposition steps are then connected by merging common peaks. In doing so, current ellipse peak parameters are replaced by ellipse parameters of the next step, where more grainy structures are scanned if both ellipses have peak data points in common. Newly found ellipses, which do not share peak data points, are added to the peak list. Compared to the standalone GIM method, WBMBT can detect peaks enclosed in the shoulder of other peaks. The evaluation of Bader *et al.* shows that this method is highly sensitive, with only a small number of false positive classifications that can be filtered out by a clustering method when comparing a set of measurements.

**Water shed transformation (WST): **Another peak finding method for IMS data is described by Bunkowski [40]. This method is based on an approach of Wegner *et al.* [49] used for spot detection on 2D gel electrophoresis images. For further details about water shed transformation, the reader is referred to the publications of Meyer *et al.* [50] or Vincent *et al.* [51].

The intuitive underlying working principle of this method is as follows. Imagine that an IMS chromatogram image is turned upside down and interpreted as a topographical surface. This surface is flooded with water, from bottom to top, from different sources that are each located at a local intensity maximum (peak). The water will accumulate stepwise in catchment basins from higher to lower intensities, *i.e.*, the highest peaks are filled with water first. 

When the water level reaches the limit of overflow to another basin, a watershed is built which separates the basins. The overall process segments an IMS chromatogram into peaks and peak parameters similar to the aforementioned methods. However, in contrast to the other approaches, this method delivers the volume of a peak, which can then be taken into account for further analysis. Unfortunately, no well-structured assessment of this method is available so far.

#### 2.3.3. Merging Peak Sets

Enabling further analysis of differences and similarities between measurements requires a grouping of peaks that are related to the same analyte. Therefore, the determination of peak sets among all measurements, together with the unambiguous assignment of peaks to those sets, is necessary. In all peak finding and characterization approaches described here, typical peak regions serve as peak group descriptors, which are characterized by a center point, 1/K_0_ radius, and RT radius, and can also be used to identify specific analytes [29,52]. The challenge is to refer each peak to a general peak region. Unfortunately, all parameters of a peak related to a particular analyte vary among several measurements. This impedes assignment to the correct peak region, especially if there is a fluctuation in the position of maximum peak intensity. Based on the coordinates of the maximum for each peak, a pipeline of two separate clustering methods is established [39]. The first clustering is used to find the mean position of each cluster. The second clustering method takes these positions as starting points and associates each peak to a cluster. Bader *et al.* [39] compared different combinations of clustering methods. The combination of Ward’s clustering method [53] (1) with a k-means algorithm using the Euclidean distance; (2) gives the best results according to the variance ratio criterion and average silhouette width [39]. 

### 2.4. Database

Driven by the demand for rapid data analysis and biomarker discovery, there is a need for the establishment of a centralized data repository to facilitate the identification of analytes and for data mining in studies such as clinical trials. In contrast to other techniques like mass spectrometric data, for which a couple of data organizing tools are available (for example OpenMS [54]), the IMS community lacks such a system.

In 2007, Lesniak developed the first database schema to organize IMS data [55]. Nevertheless, this approach is not able to store arbitrary entities, attributes and values, as well as relations between entities, which is beneficial to make the database adaptable to any kind of up-coming medical annotation data. The goal is to allow a flexible management of analytical data (MCC/IMS, GC/MS) combined with medical data (diseases, medication, age, gender, etc.) provided by physicians or biologists. Flexibility can be ensured by an ontology-based generalized data structure, which enables the system to store any kind of information without changing the database schema.

Note that a major drawback of such a generic model is a performance loss under particular circumstances due to a higher model complexity. Compared to conventional database schemas, queries are more complex and especially attribute-centric queries perform worse [56]. However, in a proper case of application, profit can be derived from the flexibility offered by such a model. 

A database system including metabolite profiles and corresponding patient data will allow the extraction of interesting data sets as a starting point for statistical analysis (Section 3) and statistical learning (Section 4). Such a system is currently being developed at KIST Europe and the Max Planck Institute for Informatics, Saarbrücken Germany.

## 3. Statistical Analysis

### 3.1. Statistical Tests

The aim of clinical diagnostics is to detect molecules, so-called biomarkers, that can give the answer to a specific question, e.g., concerning the health status of a person (disease, no-disease). If a single molecule or antibody in the blood, urine or breath having direct relation to the analyzed disease status exists, it can be detected by statistical tests. 

For methodological reasons, the assumption that the intensity of molecules within the chromatogram is drawn from a Gaussian distribution is not valid [57]. Therefore the community of ion mobility spectrometry relies on non-parametric tests, especially the Mann-Whitney U test. This test, also called rank sum test, is an unpaired hypothesis test to verify whether two samples are drawn from the same distribution, or more precisely, whether one of two samples of independent observations tends to have smaller values than the other. In the following we briefly describe several studies analyzing exhaled air of patients using MCC/IMS, applied Mann-Whitney U test to distinguish between different health states.

In 2011, Bessa *et al.* [58] and Koczulla *et al.* [59] examined the volatile organic compounds in exhaled breath of patients suffering from chronic obstructive pulmonary disease (COPD). The first study used the rank sum test to differentiate between COPD patients and healthy controls. The second study focused on the classification of COPD patients with and without alpha 1-antitrypsin deficiency. In both studies, rank sum analysis led to the identification of potential single biomarker candidates.

A further example for the application of the rank sum test is the detection of microorganisms in the human body. According to Rabis *et al.* 2011, bacteria produce VOCs [60]. In their study, they focused on *pseudomonas aeruginosa*, a bacterium, which is associated with COPD exacerbation. They investigated the exhaled air of *pseudomonas*-infected patients compared to healthy non-smokers. The Mann-Whitney U test was applied to deduce the most discriminative signals, which resulted in a best accuracy of 88%.

### 3.2. Correlation

Another method to evaluate the relation of different variables is the Pearson correlation. In 2011 Maddula *et al.* utilized this to analyze the similarity between the metabolites, which they used as indicators in their disease analysis, especially those identified as important [61]. In fact, the Pearson correlation can be used (1) to find sets or clusters of related metabolites exposing the same behavior and (2) to reduce the data set by selecting representatives for each cluster. Moreover, it would be interesting to investigate whether a set of metabolites showing the same pattern also originates from the same pathway.

Additionally, the Pearson correlation was applied to evaluate whether medication levels detected within the breath can be associated with other state of the art methods for that purpose. Both Kreuder *et al.* [62] and Carstens *et al.* [63] investigated the intensity of propofol within the breath of patients undergoing anesthesia, utilizing the MCC/IMS technique. While Kreuder *et al.* [62] compared the MCC/IMS propofol intensity with the TCI pump calculated plasma values, Carstens *et al.* [63] correlated their results with the propofol serum concentration determined by gas chromatography-mass spectrometry (GC/MS). Both studies report a strong correlation between the level of propofol in breath and the used plasma level.

### 3.3. Principal Component Analysis

Principal Component Analysis (PCA), first introduced by Pearson [64], is a computational/-mathematical technique that is used in practice to reduce the dimensionality of a given data set and to find linearly independent variables that most dominantly express the underlying data model (principal components). A very high dimensional of the data set can cause extensive over-fitting in any following statistical learning procedures. A prior reduction of the parameter space is necessary. In contrast to other methods that use an orthogonal transformation, a PCA allows for an inverse transformation of the data back into the initial parameter space. Therefore, PCA results can directly be interpreted and used for follow-up analyses. These characteristics are fundamental in the statistical analysis of metabolic measurements derived from IMS breath sampling. Westhoff *et al.* applied a PCA for the detection of distinctive metabolites that separated breath samples taken from COPD patients from those of healthy controls [65]. The authors identified a single discriminative analyte (cyclohexanone, CAS 108-94-1) out of 104 initially detected VOCs that classified the datasets with a sensitivity of 60%, a specificity of 91%, and a positive predictive value of 95%. Note that further studies still have to validate this analyte as a COPD indicator. In a related study, Westhoff *et al.* embedded PCA into a comprehensive statistical analysis of IMS breath samples taken from 95 COPD patients and 35 healthy individuals including Mann-Whitney U test, correlation analysis and decision trees [66]. Cheung *et al.* applied PCA to Py-GC-DMS data sets of two strains of *B. subtilis* and one strain of *B. megaterium* [67]. PCA proved to be sufficient to discriminate bacterial strains on species level, while separation of the two *B. subtilis* strains required chemometric methods using supervised classification. Additionally, the PCA algorithm results in a multidimensional scaling of the original data by using the first two or three principal components. This can be visualized by various software tools or packages for the statistical environment R [68]. 

## 4. Statistical Learning

### 4.1. Reduced Ion Mobility Prediction

Initially, the ion mobility spectrometry method has been used to detect specific target analytes with known reduced ion mobility K_0_. This changed during the shift of application areas from military towards medical and process control purposes resulting in the analysis of complex mixtures in rather humid environment. Therefore, an accurate database of relevant analytes and their specific positions (K_0_, retention time) for automated identification of the peaks within an IMS chromatogram is needed. To create such a database either each reference substance has to be measured by the IMS or parallel measurements with other mass spectrometric methods have to be carried out. However, to create a reference database solely on the basis of these methods is time consuming and expensive. Therefore, computational methods for reduced ion mobility prediction have been developed. 

An early review by Revercomb and Mason describes the fundamental theory of ion mobility (K) on a molecular scale [69]. According to this theory, K can be calculated using several factors: charge of the ion, number density of the drift gas, the Bolzmann constant, temperature, ion mass, mass of the drift gas, and the ion collision cross section. Considering constant operating conditions and only single charged positive molecular ions, K as well as K_0_ is only dependent on ion mass and collision cross section. For homologous series of compounds the correlations between ion mobility and ion mass are fairly accurate, which was demonstrated by studies on ketones and amines in the late seventies [70,71]. 

The first attempts to predict K_0_ for non-homologous compounds incorporated structural descriptors in addition to the mass, to encode the elusive collision cross section of the fundamental ion mobility theory. Quantitative structure-property relationship methods, namely multiple linear regression and computational neural networks in combination with numeric structural features were utilized to predict the mobility. The set of features consisted of topological, geometric, electrostatic, and combinations of the three types of molecule attributes.

The validation on a test-set resulted in a root mean square error of around 0.04 [72]. A similar set of functional descriptors was used in 2007 in combination with a multiple linear regression for feature selection and a projection pursuit regression for prediction [73].

A more recent approach utilized the information of the number of carbon atoms within the molecule, to predict the reduced ion mobility of polar aliphatic organic compounds. In this study they discovered a linear relation between the number of carbon atoms and the reduced ion mobility of the compounds within a certain homologous series, as a fast and intuitive method for K_0_ estimation [74].

### 4.2. Probabilistic Relational Learning

Probabilistic relational learning (PRL) is a very active field in research at the intersection of machine learning, logic, and probability theory. Relational models are the most common representation of structured data. Most statistical learning methods work with “flat” data representations, including objects and their attributes. In contrast to this, probabilistic relational models allow the properties of objects to depend probabilistically on each other and on the properties of other related objects. Data as enterprise business information, marketing and sales data and medical records can be explicitly modeled as relational models to discover useful relationships and even more importantly, to discover unknown information [75]. 

Particularly the identification of biomarkers is one of the major goals in clinical diagnostic research. A biomarker or a set of biomarkers in clinical breath diagnostics is a VOC or a set of VOCs, of which the presence, absence or intensity is an indicator for a certain disease. Therefore statistical relational learning is well suited for analyzing the structure of IMS data and for identifying biomarkers.

In IMS research, the methodology of probabilistic relational learning was applied in 2011 for the first time by Finthammer *et al.* for biomarker detection and biomedical diagnosis of bronchial carcinoma [76]. First, they applied a k-means algorithm for peak clustering in order to identify the molecules detected by the MCC/IMS. Subsequently, they estimated the probability of a peak cluster (PCi) occurring in a measurement and the conditional probability, *i.e.*, the probability for a measurement including a certain peak PCi originating from a patient suffering from bronchial carcinoma. Based on these probabilities, a set of diagnostic rules for bronchial carcinoma was learned by using Inductive Logic Programming [77]. The result is a Markov logic network (MLN) defined as a set of (positive or negative) weighted first-order logic formulas together with a set of constants. Table 1 shows an example of the resulting diagnostic rules.

**Table 1:** Example of MLN formula emerged from Alchemy’s structure learning (90% accuracy), where *pc i (M )* is the presence of peak cluster number *i* in sample *M* and *bc ( M )* indicates that the sample *M* originates from a patient suffering from bronchial carcinoma (*¬bc ( M )* = healthy control) [76]. Reproduced with permission from Finthammer *et al.*, International Journal of Ion Mobility Spectrometry published by Springer-Verlag, 2010.

#	Formula	Weight
37	*pc7(M )* *⇒ bc ( M )*	4.43
39	*pc11 (M )* *⇒ pc9(M )*	4.82
44	*pc17 (M )* *∧ pc28 (M )* *⇒ pc21 (M )*	5.05
46	*pc15 (M )* *∧ pc25 (M )* *⇒ pc5(M )*	−4.30
47	*pc17 (M )* *∧ pc19 (M )* *∧ pc20 (M )* *⇒ pc9(M )*	−8.98
53	*pc12 (M )* *∧ pc20 (M )* *∧ pc22 (M )* *⇒ pc11 (M )*	−8.14
57	*¬pc1(M )* *∧¬pc18 (M )* *∧¬pc23 (M )* *∧ pc31 (M )* *⇒ bc ( M )*	6.38
61	*¬pc10 (M )* *∧ pc14 (M )* *∧¬pc18 (M )* *∧ pc21 (M )* *⇒ bc ( M )*	7.15
62	*¬pc12 (M )* *∧¬pc22 (M )* *∧¬pc30 (M )* *∧ pc31 (M )* *⇒ bc ( M )*	7.49
66	*pc4(M )* *∧ pc26 (M )* *∧ pc28 (M )* *∧ pc29 (M )* *⇒ bc ( M )*	−5.62
68	*¬pc9(M )* *∧¬pc13 (M )* *∧¬pc16 (M )* *∧ pc23 (M )* *∧¬pc29 (M )* *⇒ ¬bc ( M )*	4.01
70	*pc1(M )* *∧ pc3(M )* *∧¬pc15 (M )* *∧¬pc23 (M )* *∧ pc26 (M )* *⇒ ¬bc ( M )*	−5.18
72	*pc0(M )* *∧¬pc11 (M )* *∧¬pc12 (M )* *∧¬pc21 (M )* *∧ pc22 (M )* *⇒ ¬bc ( M )*	2.45
75	*pc5(M )* *∧ pc7(M )* *∧¬pc28 (M )* *∧¬pc29 (M )* *∧ pc31 (M )* *⇒ ¬bc ( M )*	−2.78
80	*pc0(M )* *∧¬pc12 (M )* *∧¬pc16 (M )* *∧ pc30 (M )* *∧¬pc32 (M )* *⇒ bc ( M )*	−5.55
81	*¬pc6(M )* *∧¬pc13 (M )* *∧¬pc28 (M )* *∧ pc31 (M )* *∧ pc32 (M )* *⇒ ¬bc ( M )*	5.61
82	*¬pc3(M )* *∧¬pc4(M )* *∧ pc25 (M )* *∧¬pc28 (M )* *∧¬pc32 (M )* *⇒ ¬bc ( M )*	8.77
89	*¬pc3(M )* *∧¬pc11 (M )* *∧ pc13 (M )* *∧¬pc17 (M )* *∧¬pc31 (M )* *⇒ ¬bc ( M )*	−5.15

The best model in this study was based on the rules shown in Table 1. Estimating the influence of connected peaks regarding the health state achieved a cross validation accuracy of up to 90%. However, MLN learning aims at extracting relational, intelligible information from the observed data, which can hardly be measured by statistical parameters. Nevertheless, the authors state that although the PRL allows drawing some conclusions between the occurrence of peak clusters and bronchial carcinoma, they emphasized that MLNs have certain shortcomings. From the knowledge representation point of view, the weights of a MLN formula have no clear probabilistic semantics and hence only “simple” MLNs are interpretable [76].

### 4.3. Statistical Learning and Biomarkers

The general aim of statistical learning is to use the information on the attributes of the samples, encoded in variables or features to infer a certain class. The most common application area of learning methods in bioinformatics is disease prediction. The physician gathers information on both patients suffering from a certain disease and a set of healthy controls. This information is extracted by using different diagnostic techniques, e.g. analyzing blood, urine or breath of the patients, and subsequently used to train computational models.

The first study using statistical learning approaches on a set of IMS chromatograms to predict the health status of patients was done by Baumbach *et al.* in 2007 [78]. They developed a software architecture that analyzed the data of lung cancer patients and healthy persons. Due to a lack of advanced pre-processing and peak location methods, they applied a Gaussian filter to reduce the effect of background noise. The complexity of the model was reduced by decreasing the number of features; the relevant part of each chromatogram (right of the RIP) was separated by a grid, while each feature was calculated as the average intensity of the corresponding grid element. Subsequently, a set of techniques, namely naive Bayes, multi-layer perceptron, and support vector machine (SVM) were applied to achieve an outstanding performance (accuracy and AUC both 99%) distinguishing between the healthy and the diseased [78]. Despite the good results, one has to consider that (1) the prediction was done on a comparatively large feature set and a small sample size (35 lung cancer patients and 72 healthy controls), and (2) the accuracy and AUC were evaluated on the training set.

The next study was carried out only recently in 2011 by Westhoff *et al.* [66]. This study incorporated the advanced pre-processing and peak location methods described in Section 2.3 to extract those positions in the chromatogram that correspond to volatile organic compounds. They utilized Mann-Whitney U tests and a decision tree to classify the measurements of 95 COPD patients and 35 healthy controls. Similar to the previous study, the models were trained on the whole set resulting in a very good performance (accuracy 95%). Additionally, Westhoff *et al.* extracted the most interesting molecules either having the lowest p-values by Mann-Whitney U test or chosen as variables in the decision tree [66].

Another, more recent study by Hauschild *et al.* in 2012 focused on the classification and biomarker identification of COPD and bronchial carcinoma based on MCC/IMS data. The data set was composed of 35 healthy controls (HC) and 84 patients either suffering from chronic obstructive pulmonary disease (COPD) or both COPD and bronchial carcinoma (COPD+BC). Like the study of Westhoff *et al.* [66], the advanced pre-processing and peak location methods provided by the VisualNow software were used to build the features for COPD and bronchial carcinoma prediction. To get a broad overview of the potential of the data and the different classification techniques, six different sophisticated statistical learning methods have been applied: Decision trees, naive Bayes, neural networks, random forest and linear as well as radial SVM [79]. Similar to the previous studies of Baumbach *et al.* [78] and Westhoff *et al.* [66], the set of samples was small (119 volunteers), which leads to a very noisy estimation of the predictive performance. Therefore, in contrast to the previous studies, the authors used cross validation to provide an accurate estimate for the actual performance of the predictive model. The random forest was reported to produce the best prediction results for the COPD prediction (accuracy 94%, AUC 92%) and the bronchial carcinoma prediction (accuracy 79%). Due to the low performance, the authors suggest that further analysis of the separation between COPD and bronchial carcinoma patients is needed. In fact, all tested methods showed a very low sensitivity for the COPD class in contrast to a high sensitivity for the BC class, which indicates that the differentiation between class COPD and COPD + BC is difficult. In fact, most of the measurements of COPD patients falsely predicted suffering from both COPD and bronchial carcinoma, which might be reducible to the characteristic of COPD as a common and important independent risk factor for lung cancer. Both studies, Westhoff *et al.* 2011 [66] as well as Hauschild *et al.* [79], identified a set of ten most informative features, whereby five of these twenty features overlapped in the inverse drift time as well as retention time, which means they represent the same VOCs. 

**Table 2:** An overview of the four studies in Section 4, analyzing MCC/IMS data of different diseases (bronchial carcinoma (BC) and chronic obstructive pulmonary disease (COPD)). The ACC is the accuracy given by the percentage of correctly classified samples, # is the number of samples in that study, the AUC is the area under the receiver operating characteristics (ROC) curve, and CV indicates whether cross validation was used.

Study	Disease	#	ACC	AUC	CV
Finthammer *et al.* 2010 [76]	BC	158	90%	-	√
Baumbach *et al.* 2007 [78]	BC	107	99%	99%	-
Westhoff *et al.* 2011 [66]	COPD	130	94%	-	-
Hauschild *et al.* 2012 [79]	COPD and BC	119	94%	92%	√

## 5. Summary and Conclusion

We conclude that MCC/IMS coupled with sophisticated computational methods has the potential to successfully address a broad range of biomedical questions. The investigation of a rather large number of volatile metabolites, especially in breath, opens the way for a totally non-invasive method of investigation with respect to systems biology and personalized medicine. Based on the chemical analysis of some mL of volatiles, time series and continuous investigations of cells, organs, animals and humans could be realized. Such continuous sampling is rather complicated using techniques that require the invasive collection of blood or other material. On one hand, detection limits down to the pg/L-range (ppt_v_-range) are achieved in a rather humid and complex environment. On the data analysis side, many problems on the way to a comprehensive framework for the analysis of MCC/IMS chromatograms have been solved, for example, the definition of the data format, the visualization and the general evaluation using statistical techniques. Nevertheless some computational challenges remain, Table 3.

One of the main tasks is to develop a flexible and comprehensive centralized data repository, which is still unsolved. In contrast to this, pre-processing methods like RIP detailing, smoothing and de-noising have been studied extensively in the last five to ten years. However, the assessment of the quality of these techniques has been done solely by visual appearance. The same holds for the results of the peak detection methods, which were evaluated, if at all, using visual comparison with the manually selected peak lists. Besides, the question of when a peak is recognized as a peak is still open and varies from operator to operator. In the future, pre-processing and peak picking methods have to be validated and compared according to the aim of the study, *i.e.*, the classification performance.

**Table 3:** This shows the ranking of the achievements in MCC/IMS data analysis using computational methods.

Computational requirements	Completed
Data format	***
Visualization	***
Pre-processing methods	**
Peak detection methods	**
Centralized data repository	*
Statistical approaches	***
Statistical learning methods	*
Differentiation of diseases, infections, cancer, etc.	*
Disease pathway identification	-

“***”accomplished; “**” almost complete; “*” first steps have been made; “-” not solved

The next steps in the framework would be biomarker identification and disease classification using statistical methods like Mann-Whitney U tests and more sophisticated learning methods. In contrast to the statistical analysis methods, statistical learning methods train models that are able to predict the class of samples using a set of features found to be most informative for this model. So far, the statistical learning methods applied to MCC/IMS data concentrated solely on supervised methods. However, unsupervised learning methods in general have a great potential of extracting additional information from the data, which has not been shown so far in the present field of interest. Unsupervised clustering methods can find groups of molecules or samples without any information on the classes (healthy vs. disease). Therefore, such methods are to be applied on large datasets generated in multicenter studies in different hospitals and should show their potential with respect to specific and unspecific metabolic questions in the future. To complete the framework, an automated analysis of the IMS data, starting with a centralized database, followed by an automated biomarker identification and classification, is needed, which is generally open for different instrumentations and metabolic questions.

Such a centralized and flexible storage, which is capable of gathering all kinds of confounding factors, diet or medication, for instance, allows for the discovery of their influence within the IMS chromatograms and hence a more accurate disease modeling accounting for these contaminations. One question has been left untouched so far: “How to find the disease-specific pathway?” Once the most interesting analytes considered as potential biomarkers are detected, the identity of these molecules is determined by mass spectrometric methods. Subsequently, the pathways within the human or bacterial organisms containing these potential biomarkers can be further investigated by novel computational techniques. In a recent review by Khatri *et al.*, these techniques for pathway analysis have been categorized into three generations of approaches: the first-generation "over-representation analysis" (ORA) approaches, the second-generation "functional class scoring" (FCS) approaches, and the third-generation "pathway topology" (PT) approaches [80].

We conclude that the basic computational methods for pre-processing, biomarker identification and disease prediction are available. However, a comprehensive framework providing a pipeline to automatically pre-process and evaluate complete sets of MCC/IMS data is still required.
